# Interaction Between Mesenchymal Stromal Cells and Tumor Cells Present in Cervical Cancer Influences Macrophage Polarization

**DOI:** 10.3390/cancers17193099

**Published:** 2025-09-23

**Authors:** Eduardo Bautista-Sebastián, Víctor Adrián Cortés-Morales, Guadalupe Rosario Fajardo-Orduña, Alberto Monroy-García, Marta Elena Castro-Manrreza, Alberto Daniel Saucedo-Campos, Marcos Gutiérrez-de la Barrera, Héctor Mayani, Juan José Montesinos

**Affiliations:** 1 Mesenchymal Stem Cell Laboratory, Oncology Research Unit, Oncology Hospital, National Medical Center (IMSS), Mexico City 06720, Mexico; e.bautista.sebastian@gmail.com (E.B.-S.); guadalupefajardo@hotmail.com (G.R.F.-O.); 2 Postgraduate in Biological Sciences, National Autonomous University of Mexico, Coyoacán, Mexico City 04360, Mexico; 3 Unidad de Investigación Médica en Inmunoquímica, UMAE Hospital de Especialidades, Centro Médico Nacional Siglo XXI, Instituto Mexicano del Seguro Social, Mexico City 06720, Mexico; v.adrian.cortes@gmail.com; 4 Immunology and Cancer Laboratory, Oncology Research Unit, Oncology Hospital, National Medical Center (IMSS), Mexico City 06720, Mexico; albertomon@yahoo.com; 5 Immunology and Stem Cells Laboratory, FES Zaragoza, National Autonomous University of Mexico (UNAM), Mexico City 04360, Mexico; elmar_ca@yahoo.com.mx; 6 Laboratorio 7 de Inmunología, Unidad de Morfofisiología y Función, FES Iztacala, Universidad Nacional Autónoma de México, Mexico City 54090, Mexico; beto000@msn.com; 7 Facultad de Medicina, Universidad Nacional Autónoma de México, Mexico City 04510, Mexico; marcosgub@gmail.com; 8 Hematopoietic Stem Cell Laboratory, Oncology Research Unit, Oncology Hospital, National Medical Center (IMSS), Mexico City 06720, Mexico; hmayaniv@prodigy.net.mx

**Keywords:** tumor-derived mesenchymal stromal cells, cervical cancer, macrophage polarization, T lymphocytes, immunomodulation

## Abstract

Background/Objectives: Macrophages with the M2 phenotype are an immune population with great relevance for tumor development. We have previously demonstrated that mesenchymal stromal cells (MSCs) from cervical cancer (CeCa-MSCs) enhance the immunomodulatory activity of CeCa cells on T lymphocytes; however, the effect of these cells on the ability of tumor cells to polarize macrophages had not been evaluated to date. Methods: To address this, we set out to analyze the effect of normal cervix (NCx) and CeCa-MSCs interacting with CeCa tumor cells (TCs) to polarize macrophages in a coculture system. Results: Our results show that macrophages from TC/NCx-MSC cocultures decreased CD163 expression. In turn, we observed that macrophages from TC/CeCa-MSC cocultures, in contrast to those in the presence of TCs/NCx-MSCs, increased the intracellular production of IDO, IL-4, and IL-10; decreased T lymphocyte proliferation; and increased the presence of soluble IL-10. Interestingly, coculture in the presence of TCs/NCx-MSCs decreased the capacity of macrophages to generate regulatory T lymphocyte populations, as well as their phagocytic capacity, and increased IL-6 secretion, unlike the coculture of macrophages in the presence of TCs/CeCa-MSCs. Our results show that TCs/CeCa-MSCs in cocultures, unlike TCs/NCx-MSCs, have a greater capacity to polarize macrophages to an M2 phenotype and that such macrophages have a greater immunosuppressive potential. Conclusions: This in vitro study suggests that intracellular communication between MSCs and tumor cells in CeCa may promote tumor growth through the polarization of macrophages with increased immunosuppressive activity.

## Simple Summary

Within tumor tissue, cancer cells communicate with the microenvironment to generate immunosuppressive cell populations that favor tumor progression. This study evaluated whether the interaction between cervical tumor cells (TCs) and mesenchymal stromal cells derived from cervical cancer (CeCa-MSCs) promotes the polarization of macrophages toward the M2 phenotype. The M2 macrophage polarization and the immunosuppressive potential of such cells were evaluated in terms of membrane markers, cytokine secretion, phagocytic capacity, the inhibition of T lymphocyte proliferation, and the generation of regulatory T cell populations. Our findings show that the interaction between TCs and CeCa-MSCs favors the M2 polarization of macrophages that show a high immunosuppressive capacity. This study suggests that cell communication between TCs and MSCs present in cervical tumors could favor tumor growth through the polarization of macrophages with high immunosuppressive potential.

## 1. Introduction

Cervical cancer (CeCa) is the cancer with the fourth-highest incidence in the female population internationally; however, it presents the second-highest incidence and mortality in the female population in countries with low or middle human development indices [1,2]. Human papillomavirus (HPV) is reported in up to 95% of cases, and tumor cells with genotypes 16 and 18 are responsible for 70% of cases worldwide [2].

Cervical tissue contains different populations of immune cells, such as T lymphocytes, NK lymphocytes, B lymphocytes, dendritic cells, and macrophages, for protection in the case of infection. However, when tumor cells are present, they can reprogram immune system cells to facilitate evasion of the immune response and favor tumor progression through the secretion of molecules such as IL-8, IL-4, IL-13, and IL-10, which have an immunosuppressive effect on the activation of T and NK lymphocytes [3,4], and of M-CSF, IL-10, and TGF-β, among other molecules, which promote the M2 polarization of macrophages; these cells show increased CD163 expression and intracellular IL-10 production [5,6]. Tumor-associated macrophages (TAMs) represent up to 50% of the immune population and 80% of the stromal cells within the tumor microenvironment [7,8]. TAMs are characterized by the secretion of IL-4 and IL-10, preventing the production of IL-12, decreasing T lymphocyte proliferation, and inducing the conversion of CD4+ T cells into regulatory T cells, and they can also phagocytose apoptotic cells [5,9,10,11,12,13]. The presence of a higher number of M2 macrophages is associated with a poor prognosis in patients with CeCa [14].

Mesenchymal stromal cells (MSCs) were defined by the International Society for Cell and Gene Therapy as plastic adherent cells that express CD105, CD73, and CD90; lack the expression of hematopoietic and endothelial markers; and possess the ability to differentiate to osteoblasts, adipocytes, and chondroblasts. They also possess immunoregulatory potential, and this may vary depending on the tissue of origin [15]. MSCs are part of the tumor microenvironment; previous studies indicate that they have the capacity to favor the proliferation, migration, and invasion of tumor cells, generating an optimal microenvironment for tumor progression [16,17]. Our group has reported that MSCs derived from CeCa [18] and pituitary tumors [19] favor the M2 phenotype of monocyte-derived macrophages in vitro, a capacity that is diminished in MSCs derived from non-tumor tissue. On the other hand, we have demonstrated that molecules secreted by MSCs from bone marrow increase the expression of IDO and TGF-β in tumor cells, which favors the generation of regulatory T lymphocytes [20]. Furthermore, we have reported that CeCa-MSCs secrete IL-10 [21] and TGF-β [22], which regulate tumor cells, decreasing the expression of HLA-I and thereby preventing CD8 T lymphocyte cytotoxicity. All these studies indicate that MSCs and tumor cells (TCs) interact closely, giving the latter a growth advantage and thus favoring their development.

Based on the above, in the present work, we decided to investigate how the interaction of CeCa-MSCs and CeCa tumor cells (TCs) influences macrophage polarization towards the M2 phenotype.

## 2. Materials and Methods

### 2.1. Culture of Mesenchymal Stromal Cells (MSCs)

MSCs obtained from CeCa (n = 5) and NCx (n = 5) biopsies that were previously cryopreserved and characterized [18,21] were used in the experiments. Briefly, as we previously reported [18], thawed MSCs were cultured in T75 flasks (Corning, Corning, NY, USA) in the presence of Lg-DMEM (Gibco BRL, Rockville, MD, USA) supplemented with 10% fetal bovine serum (FBS; Gibco BRL) and collected at 80% confluence. MSCs were used at passages 4–5 for the experimental assays.

### 2.2. Culture of Tumor Cell Lines

Tumor lines derived from patients with cervical cancer, CaSki (HPV positive) and C33A (HPV negative), were used for the experiments. The tumor lines were cultured in 75 cm^2^ flasks (Corning) with RPMI-1640 culture medium (Roswell Park Memorial Institute 1640, Gibco) supplemented with 10% FBS, 10 μL/mL penicillin–streptomycin, 10 μL/mL gentamicin, and 10 μL/mL L-glutamine. When they reached 80% confluence, they were detached with 0.75X trypsin for experimental assays.

### 2.3. Obtention of Peripheral Blood Mononuclear Cells (PBMCs)

Peripheral blood mononuclear cells (PBMCs) were obtained from healthy donors using a density gradient (Lymphoprep, STEMCELL TECHNOLOGIES, Vancouver, BC, Canada). The PBMCs were seeded in RPMI 1640 supplemented with 10% FBS, 10 μL/mL penicillin–streptomycin, 10 μL/mL gentamicin, and 10 μL/mL L-glutamine until use.

### 2.4. Obtention of Monocytes

Monocytes were obtained from PBMCs by enrichment with human microbeads and MACS MS columns (Miltenyi Biotec, Bergisch Gladbach, Germany), recovering the CD14+ CD16- negative fraction. The monocytes were seeded in RPMI 1640 supplemented with 10% FBS, 10 μL/mL penicillin–streptomycin, 10 μL/mL gentamicin, and 10 μL/mL L-glutamine until use.

### 2.5. Obtention of CD3+/CD4+ T Lymphocytes

CD3+ or CD4+ T lymphocytes were obtained from PBMCs by enrichment with microbeads and human MACS MS columns, recovering the CD3+ or CD4+ positive fraction. T lymphocytes were seeded in RPMI 1640 supplemented with 10% FBS, 10 μL/mL penicillin–streptomycin, 10 μL/mL gentamicin, and 10 μL/mL L-glutamine until use.

### 2.6. Evaluation of Membrane Markers in Macrophages

To determine the effect of the presence of TCs/MSCs on macrophage polarization, cell contact cocultures of TCs/MSCs/monocytes (in a 1:1:5 ratio) were performed in 24-well plates (Corning) for 72 h in RPMI/DMEMlg/FBS medium (45%/45%/10%) supplemented with antibiotics and L-glutamine. Cells were collected and washed with 1X phosphate-buffered saline (PBS). The cells were stained with Ghost Dye Red 780 marker according to the manufacturer’s instructions (TONBO biosciences, San Diego, CA, USA). They were then blocked with FBS for 15 min at 4 °C and washed again with PBS. Subsequently, antibodies were added to evaluate the expression of extracellular membrane markers—Pacific Blue anti-human CD45 (Biolegend, San Diego, CA, USA), phenotype M1: PE anti-human CD80 (BD Bioscience, Franklin Lakes, NJ, USA), APC anti-human CD86 (BD Bioscience), and PE-Cy7 anti-human HLA-DR (Biolegend), as well as phenotype M2: Brillant Violet 510 anti-human CD14 (Biolegend), FITC anti-human CD163 (Biolegend), and PE-Cy5 anti-human CD206 (Biolegend)—for 20 min at 4 °C. Finally, the cells were fixed with 1% paraformaldehyde for 10 min at 4 °C and washed with PBS. Acquisitions were performed on the Aurora spectral flow cytometer (Cytek Biosciences, Fremont, CA, USA) and analyzed with FlowJo V10.10 software (Ashland, Wilmington, DE, USA). Macrophages cultured without TCs/MSCs were used as a control.

### 2.7. Assessment of Macrophage Phagocytic Capacity

To determine the effect of TCs/MSCs on the phagocytic capacity of macrophages, TCs/MSCs/monocytes (in a 1:1:5 ratio) were cocultured in 96-well plates with a Transwell system (0.4 μm) (HTS Transwell-96, Corning). TCs/MSCs were seeded on the Transwell, and macrophages in the lower zone, for 72 h; subsequently, the membrane was removed and then the medium was removed to add RPMI 1640 with pH Rodo Green *E. coli* BioParticles Conjugate (Invitrogen, Waltham, MA, USA) at a 1:10 dilution for 45 min at 37 °C. The cells were collected and washed with phosphate-buffered saline (PBS) 1X. For detection, the antibodies Pacific Blue anti-human CD45 and PE-Cy7 anti-human HLA-DR were then added for 20 min at 4 °C. Acquisitions were performed on the Aurora spectral flow cytometer and analyzed with FlowJo V10.10 software. Macrophages cultured without TCs/MSCs were used as a control.

### 2.8. Detection of Intracellular IDO, IL-4, and IL-10 Molecules in Macrophages

To determine the effect of TCs/MSCs on the generation of intracellular molecules in macrophages, direct contact cocultures of TCs/MSCs/monocytes (in a 1:1:5 ratio) were performed in 24-well plates for 72 h in RPMI/DMEMlg/FBS medium (45%/45%/10%) supplemented with antibiotics and L-glutamine. They were incubated for 5 h with an intracellular protein transport inhibitor (BD Bioscience) according to the manufacturer’s instructions. Cells were collected and washed with 1X PBS, and then 25 μL of Ghost Dye Red 780 viability marker (TONBO Biosciences; 1 μL of stock diluted in 1499 μL of PBS) was added for 15 min at room temperature, and the mixture was washed with PBS. Cells were blocked with FBS for 15 min at 4 °C and washed again with PBS. After blocking, Pacific Blue extracellular anti-human CD45 antibody was added for 20 min at 4 °C, and the cells were then washed. The cell membrane was then fixed and permeabilized with the FOXP3 permeabilization kit according to the manufacturer’s instructions (Invitrogen), and antibodies to detect intracellular markers—Brilliant Violet 421 anti-human IL-10 (Biolegend), PE anti-human IDO (R&D Systems, Minneapolis, MI, USA), and APC anti-human IL-4 (BD Biosciences)—were added for 30 min at 4 °C, before washing with PBS. Acquisitions were performed on the Aurora spectral flow cytometer and analyzed with FlowJo V10.10 software. Macrophages cultured without TCs/MSCs were used as a control.

### 2.9. T Lymphocyte Proliferation Inhibition Capacity of Macrophages

To evaluate the effect of TCs/MSCs on the ability of macrophages to decrease T lymphocyte proliferation, TC/MSC/macrophage cocultures (in a 1:1:5 ratio) were performed in 96-well plates with the Transwell 0.4 μm. TCs/MSCs were placed on top, and macrophages were placed on the bottom for 72 h; subsequently, the Transwell was removed, and the generated macrophages were cocultured with CD3+ T lymphocytes that were previously labeled with 2.5 μM of carboxyflurescein succinimidyl (CFSE, Thermo Fisher Scientific, Waltham, MA, USA) in direct contact in a 1:1 ratio, and anti-CD2/CD3/CD28 beads (T Cell Activation/Expansion Kit, Miltenyi) were added as an activator. After 4 days of coculture, non-adherent cells were collected and washed with PBS. Viability staining was performed with 7AAD (BD Bioscience) according to the manufacturer’s instructions. The cells were blocked with FBS for 15 min at 4 °C and washed with PBS. Subsequently, the extracellular antibodies APC anti-human CD3 (BD Biosciences), PE-Cy5 anti-human CD4 (BD Bioscience), and PE anti-human CD8 (BD Bioscience) were added for 20 min at 4 °C, and finally, the cells were washed with PBS. Acquisitions were performed on an Aurora spectral flow cytometer and analyzed with FlowJo V10.10 software. Activated T lymphocytes cultured in the absence of macrophages were considered controls.

### 2.10. Capacity of Macrophages for Generation of Regulatory T Lymphocytes

To determine the effect of TCs/MSCs on the capacity of macrophages to generate regulatory T lymphocytes, cocultures of TCs/MSCs/monocytes (in a 1:1:5 ratio) were performed in 96-well plates with the 0.4 μm Transwell system. TCs/MSCs were placed on top and macrophages on the bottom for 72 h; then, the Transwell was removed, the CD4+ T cells were cocultured with macrophages in a 1:1 ratio in direct contact, and anti-CD2/CD3/CD28 beads (T Cell Activation/Expansion Kit, Miltenyi) were added as activators. After six days of coculture, the non-adherent cells were collected and washed with PBS. A 25 μL volume of Ghost Dye Red 780 viability marker (TONBO Biosciences; 1 uL of stock diluted in 1499 μL of PBS) was added for 15 min at room temperature, and the cells were then washed with PBS. The cells were blocked with FBS for 15 min at 4 °C and washed again with PBS. After blocking, extracellular PE-Cy5 anti-human CD4 antibody and FITC anti-human CD25 antibody (BD Bioscience) were added for 20 min at 4 °C, and the cells were then washed. The cell membrane was then fixed and permeabilized with the FOXP3 permeabilization kit according to the manufacturer’s instructions (Invitrogene), and anti-human FOXP3 PE antibody was added for 30 min at 4 °C; finally, the cells were washed with PBS. Acquisitions were performed on the Aurora spectral flow cytometer and analyzed with FlowJo V10.10 software. The generation of Treg lymphocytes in the absence of macrophages was considered a control.

### 2.11. Evaluation of Cytokines Present in the Supernatant

To determine the concentration of soluble molecules secreted into the medium, the supernatants of the cocultures were obtained and stored at −70 °C until further use. The cytokines present were identified using M1/M2 macrophage polarization panel kit cytometry beads (BioLegend), following the supplier’s instructions. Acquisitions were performed on the FACS Aria II flow cytometer (BD Biosciences). The data obtained were analyzed with the free LEGENDplex BioLegend software (https://legendplex.qognit.com (accessed on 20 June 2024)).

### 2.12. Statistical Analysis

Statistical analysis was performed in GraphPad Prism 9 software. Comparisons between groups were performed using the Kruskal–Wallis test followed by the Mann–Whitney U post hoc test. Values of *p* < 0.05 were considered significant.

## 3. Results

### 3.1. NCx-MSCs in Contrast to CeCa-MSCs Decreased the CD163 Expression on Macrophages Cocultured with the CaSki Cell Line

It has been reported that tumor cells can increase the expression of M2 markers such as CD163 [6,23,24], as well as decrease M1 markers such as CD86 in vitro, an effect that is more evident if the tumor cells come from an aggressive-stage cancer [25], which promotes tumor development. In this study, we analyzed the effect derived from the interaction of CeCa-TCs and MSCs (TCs/MSCs) from NCx and CeCa on the expression of M1 or M2 markers in macrophages. Individual experiments of macrophages cocultured in cell contact for 3 days with TCs (C33A and CaSKi cell lines) and MSCs derived from NCx and CeCa were compared to macrophages cultured in the absence of TCs/MSCs with regard to the intensity of the positive expression of membrane molecules, measured as the mean fluorescence intensity (MFI), characteristic of M1 or M2 polarization. No difference was observed between the groups with regard to the percentage of positive cells.

**Figure 1:**
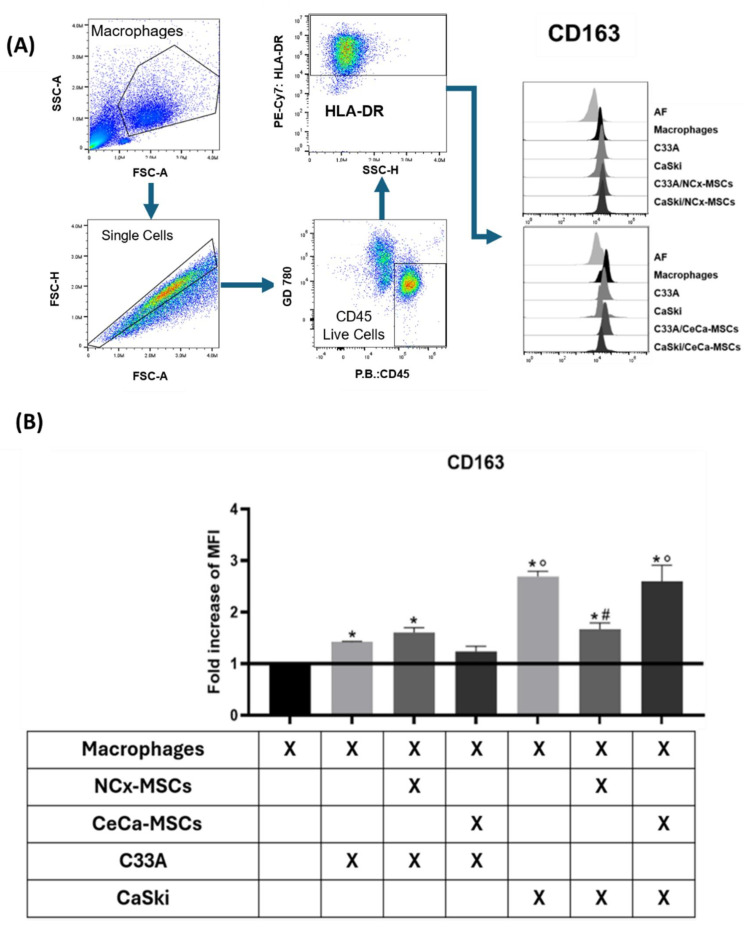
Macrophages from cocultures with CaSki/NCx-MSCs lowered the CD163 expression. To evaluate macrophage polarization toward an M2 phenotype, monocytes were cocultured for 3 days with tumor cells (C33A and CaSKi, cervical cancer cell lines) and MSCs derived from NCx and CeCa, and CD163 expression on the macrophages obtained was quantified by flow cytometry. (**A**) Flow cytometry selection strategy. Representative hierarchy of a sample starting from a population of macrophages obtained from coculture selecting CD45+HLA-DR+ populations. (**B**) Bar graphs of the fold increase relative to macrophage control for the M2 marker upon coculture with TCs or TCs/MSCs. Bar graphs represent the means with standard errors (n = 5). The black line indicates the control for comparison of the fold increase. The Kruskal–Wallis test followed by the Mann–Whitney U post hoc test was conducted. * Significant difference from control. ^#^ Significant difference from tumor line. ° Significant difference from NCx-MSCs. *, °, ^#^ Significant difference, *p* < 0.05. NCx-MSCs: normal cervix-derived mesenchymal stromal cells; CeCa-MSCs: cervical-cancer-derived mesenchymal stem cells; AF: autofluorescence; MFI: median fluorescence intensity.

When we evaluated the expression of the M1 markers CD80, CD86, and HLA-DR in macrophages, we did not observe any differences in any cocultures in the presence of TCs or TCs/MSCs. Similarly, no changes in the expression of M2 markers (CD14 and CD206) were observed in macrophages in such cocultures. In order to evaluate the expression of the CD163 marker in cocultures, we identified the macrophage population using dot plot analysis. Subsequently, we selected the singlets, from which we selected the CD45+ cells that mark a hematopoietic lineage, to later evaluate the HLA-DR+ population, a marker characteristic of antigen-presenting cell populations such as macrophages. Finally, we analyzed the expression of CD163 in this population (Figure 1A). Macrophages that were cocultured with CeCa-TCs and C33A (fold change compared to the control: 1.42 ± 0.01; *p* < 0.05) and CaSki (fold change compared to the control: 2.69 ± 0.10; *p* < 0.05) significantly increased CD163 expression regarding the control (Figure 1A,B). No difference in CD163 expression was observed in macrophages cocultivated with C33A/NCx-MSCs (fold change compared to the control: 1.60 ± 0.09) and C33A/CeCa-MSCs (fold change compared to the control: 1.24 ± 0.10). Interestingly, the expression of CD163 increased in cocultures with CaSKi/CeCa-MSCs (fold change compared to the control: 2.59 ± 0.31; *p* < 0.05) in comparison with CaSKi/NCx-MSCs (fold change compared to the control: 1.66 ± 0.10) (Figure 1A,B). These results show that NCx-MSCs decreased the CD163 expression on macrophages co-cultured with CaSki cell line in contrast to CeCa-MSCs; such a decrease was not observed with the C33A cell line.

### 3.2. NCx-MSCs in Contrast to CeCa-MSCs Decreased the Intracellular Expression of IL-4 and IL-10 in Macrophages Cocultured with the C33A Cell Line

**Figure 2:**
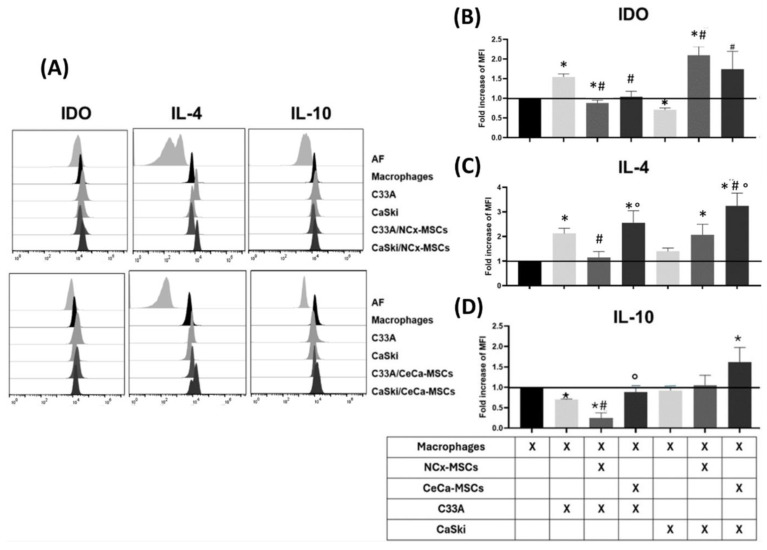
Macrophages from cocultures with TCs/CeCa-MSCs increased their intracellular expression of IL-4 and IL-10. To determine the effect of TCs/MSCs on the generation of intracellular molecules in macrophages, cocultures of TCs/MSCs/monocytes were performed for 3 days, and the expression of intracellular IL-4 and IL-10 in the macrophages obtained was analyzed by flow cytometry. (**A**) Representative histograms of the fold increase with respect to the macrophage control for intracellular expression of IDO, IL-4, and IL-10 in macrophages cocultured with TCs or TCs/MSCs. Bar graphs of the fold increase with respect to the macrophage control for intracellular expression of IDO (**B**), IL-4 (**C**), and IL-10 (**D**) in macrophages cocultured with TCs or TCs/MSCs. Bar graphs represent the means with standard errors (n = 5). The black line indicates the control to compare the fold increase in MFI. The Kruskal–Wallis test followed by the Mann–Whitney U post hoc test was conducted. * Significant difference with respect to the control. ^#^ Significant difference with respect to tumor line. ° Significant difference with respect to NCx-MSCs. *, ^#^, ° Significant difference, *p* < 0.05. TCs: tumor cells; C33A and CaSKi: cervical cancer cell lines; NCx-MSCs: normal cervix-derived mesenchymal stromal cells; CeCa-MSCs, cervical-cancer-derived mesenchymal stem cells; AF: autofluorescence; MFI: median fluorescence intensity.

M2 macrophages can express anti-inflammatory molecules such as IDO, IL-4, and IL-10 [26,27]. In CeCa, it has been reported that IL-10 is generated in macrophages, tumor cells, and other cells of the microenvironment and that HPV infection in tumor cells increases the production of this molecule, favoring tumor progression [28]. Similarly, the presence of IDO in M2 macrophages has been associated with a worse prognosis in cancer and regulation of the microenvironment towards a pro-tumor phenotype [29]. With this background, we analyzed whether the interaction between TCs and MSCs influences the intracellular expression of IDO, IL-4, and IL-10 in macrophages. To determine the effect of TCs/MSCs on the generation of intracellular molecules in macrophages, direct contact cocultures of TCs/MSCs/monocytes were performed and analyzed by flow cytometry. Our results show that coculture with C33A/NCx-MSCs (fold change compared to the control: 0.87 ± 0.07) and C33A/CeCa-MSCs (fold change compared to the control: 1.03 ± 0.14) did not increase the IDO expression in macrophages compared to C33A or the control (Figure 2A,B). However, we observed an increase in the expression of IDO in macrophages cocultured with CaSKi/NCx-MSCs (fold change compared to the control: 2.09 ± 0.22; *p* < 0.05) and CaSKi/CeCa-MSCs (fold change compared to the control: 1.73 ± 0.45; *p* < 0.05), compared to CaSki and the control (Figure 2A,B). Cocultures with C33A/CeCa-MSCs (fold change compared to the control: 2.55 ± 0.49; *p* < 0.05) increased IL-4 expression in macrophages compared to C33A/NCx-MSCs and the control. Interestingly, macrophages in cocultures with CaSKi/CeCa-MSCs increased IL-4 expression (fold change compared to the control: 3.24 ± 0.52; *p* < 0.05) compared to CaSKi/NCx-MSCs, CaSKi, and the control (Figure 2A,C). When assessing intracellular IL-10 expression in macrophages, we observed an increase in cocultures with C33A/CeCa-MSCs (fold change compared to the control: 0.88 ± 0.11; *p* < 0.05) compared to C33A/NCx-MSCs (fold change compared to the control: 0.24 ± 0.12) (Figure 2A,D) and, in a similar fashion, in the presence of CaSKi/CeCa-MSCs (fold change compared to the control: 1.62 ± 0.35; *p* < 0.05) compared to CaSKi and CaSKi/NCx-MSCs (Figure 2A,D). These results indicate that cocultures with NCx-MSCs, unlike those with CeCa-MSCs, decrease the intracellular expression of IL-4 and IL-10 in macrophages cocultured with the C33A cell line, whereas with the CaSki cell line, it was only observed for IL-4. However, we did not observe differences in IDO expression under these conditions.

### 3.3. Macrophages from Cocultures with NCx-MSCs and C33A and CaSki Cell Lines Did Not Decrease T Lymphocyte Proliferation

**Figure 3:**
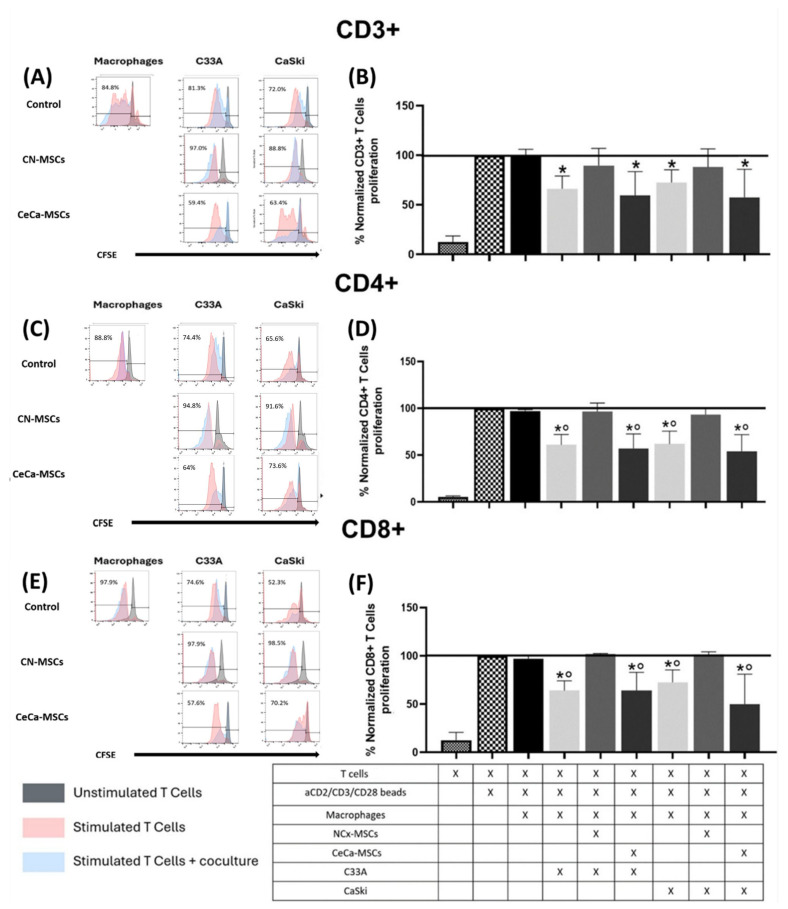
Macrophages from cocultures with TCs/NCx-MSCs did not decrease T cell proliferation. To evaluate the ability of macrophages to decrease T lymphocyte proliferation, macrophages obtained from cocultures of TCs/MSCs/monocytes were cultured with CFSE-labeled CD3+ T lymphocytes, which were quantified by flow cytometry. Representative histogram plots of the percentage proliferation of CD3+ (**A**), CD4+ (**C**), and CD8+ T lymphocytes (**E**). Bar graphs of the percentage proliferation of CD3+ (**B**), CD4+ (**D**), and CD8+ T lymphocytes (**F**). Bar graphs represent the means with standard errors (n = 5). The black line indicates the control to compare the percentage of proliferation. The Kruskal–Wallis test followed by the Mann–Whitney U post hoc test was conducted. * Significant difference with respect to the control. ° Significant difference with respect to NCx-MSCs. *, ° Significant difference, *p* < 0.05. TCs: tumor cells; C33A and CaSKi: cervical cancer cell lines; NCx-MSCs: normal cervix-derived mesenchymal stromal cells; CeCa-MSCs: cervical-cancer-derived mesenchymal stem cells; CFSE: carboxyflurescein succinimidyl.

Within the tumor microenvironment, it has been found that macrophages are programmed by tumor cells to decrease T lymphocyte proliferation [12,30,31]. This characteristic has also been observed in macrophages stimulated by MSCs with immunosuppressive capacity [31,32]; however, it is not yet known whether the interaction between both cell types could increase such capacity in macrophages. Because we found that the interaction of TCs/CeCa-MSCs increased the intracellular expression of IL-4 and IL-10 in macrophages to a greater extent than TCs/NCx-MSCs, we decided to evaluate their functional ability to inhibit T lymphocyte proliferation. To evaluate the ability of macrophages to decrease T lymphocyte proliferation, they were cocultured with CD3+ T lymphocytes labeled with CFSE, which was quantified by flow cytometry. The results show a decrease in the proliferation of CD3+ T lymphocytes in the presence of macrophages previously cocultured with TCs (C33A: 66.35% ± 12.77; CaSki: 72.59% ± 12.79) and TCs/CeCa-MSCs (C33A: 59.48% ± 24.23; CaSki: 57.57% ± 28.37), when compared to the control and TCs/NCx-MSCs (C33A: 89.63% ± 17.32; CaSki: 88.07% ± 18.51) (Figure 3A,B). Similarly, we observed a significant (*p* < 0.05) decrease in CD4+ T lymphocyte proliferation induced by macrophages that were cocultured with TCs (C33A: 61.014% ± 11.04; CaSki: 61.78% ± 13.98) and TCs/CeCa-MSCs (C33A: 57.16% ± 15.46; CaSki: 54.18% ± 17.71) compared to the control and TCs/NCx-MSCs (C33A: 96.48% ± 9.24; CaSki: 93.31% ± 6.79) (Figure 3C,D). Finally, CD8+ T lymphocyte proliferation decreased significantly (*p* < 0.05), induced by macrophages coming from cocultures with TCs (C33A: 64.10% ± 10.03; CaSki: 72.61% ± 13.00) and TCs/CeCa-MSCs (C33A: 63.99% ± 18.92; CaSki: 49.93% ± 31.10) compared to the control and TCs/NCx-MSCs (C33A: 101.80% ± 1.19; CaSki: 101.63% ± 2.61) (Figure 3E,F). These results indicate that macrophages cocultured with NCx-MSCs and C33A and CaSki cell lines, unlike those with CeCa-MSCs, do not decrease T lymphocyte proliferation.

### 3.4. Macrophages from Cocultures with TCs/NCx-MSCs Decreased Their Capacity to Induce the Generation of CD4+CD25+FOXP3+ Regulatory T Cells

**Figure 4:**
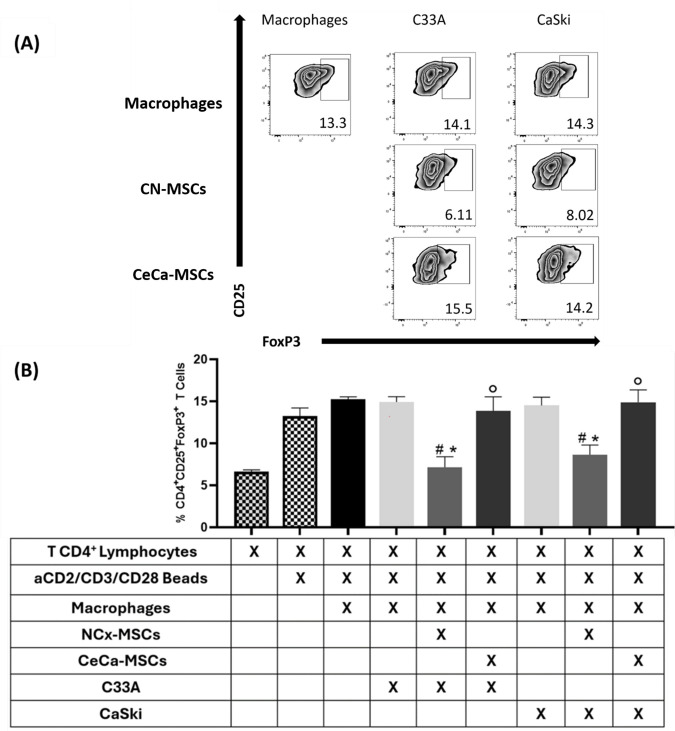
Macrophages from cocultures with TCs/NCx-MSCs lowered their capacity to generate regulatory T lymphocytes. To determine the capacity of macrophages to generate regulatory T lymphocytes, macrophages obtained from cocultures of TCs/MSCs/monocytes were cultured with CD4+ T lymphocytes, and the expression of CD25 and intracellular FOXP3 in lymphocytes was quantified by flow cytometry. (**A**) Zebra plots representative of the percentage of CD4+CD25+FOXP3+ regulatory T lymphocytes. (**B**) Bar graphs of the percentage of CD4+CD25+FOXP3+ regulatory T lymphocytes. Bar graphs represent the means with standard deviations (n = 5). The Kruskal–Wallis test followed by the Mann–Whitney U post hoc test was conducted. * Significant difference with respect to macrophage control. ^#^ Significant difference with respect to tumor line. ° Significant difference with respect to NCx-MSCs. *, ^#^, ° Significant difference, *p* < 0.05. TCs: tumor cells; C33A and CaSKi: cervical cancer cell lines; NCx-MSCs: normal cervix-derived mesenchymal stromal cells; CeCa-MSCs: cervical-cancer-derived mesenchymal stem cells.

It has been reported that M2 macrophages that interact with tumor cells can generate CD3+CD25+FOXP3+ regulatory T cells [9,23]. We have shown that MSCs enhance the ability of macrophages to generate these populations because they favor M2 polarization [18]. Our results show that, compared to TCs/NCx-MSCs, TCs/CeCa-MSCs favor the M2 polarization of macrophages to a greater extent; therefore, we decided to evaluate their capacity to generate regulatory T cells. To determine the capacity of macrophages to generate regulatory T lymphocytes, they were cocultured with CD4+ T lymphocytes, and CD25 and intracellular FOXP3 expression in lymphocytes was quantified by flow cytometry. We observed that macrophages from cocultures with TCs/NCx-MSCs significantly decreased (*p* < 0.05) the percentage of CD4+CD25+FOXP3+ regulatory T cells (C33A: 7.13% ± 1.27; CaSki: 8.63% ± 1.15; Figure 4A,B), compared to those from cocultures with TCs/CeCa-MSCs (C33A: 14.88% ± 1.47; CaSki: 13.86% ± 1.68; Figure 4A,B). These results show that NCx-MSCs lower the ability of macrophages cocultured with CaSki and C33A cell lines to generate CD4+CD25+FOXP3+ regulatory T cells and, furthermore, that macrophages obtained from cocultures with TCs/CeCa-MSCs have a greater capacity to generate such T lymphocyte populations compared to those from cocultures with TCs/NCx-MSCs, which is related to the reduced ability of TCs/NCx-MSCs to induce macrophage polarization toward the M2 phenotype (Figure 1).

### 3.5. NCx-MSCs Decreased the Percentage of Macrophages with Phagocytic Capacity Cocultured with Tumor Cell Lines

**Figure 5:**
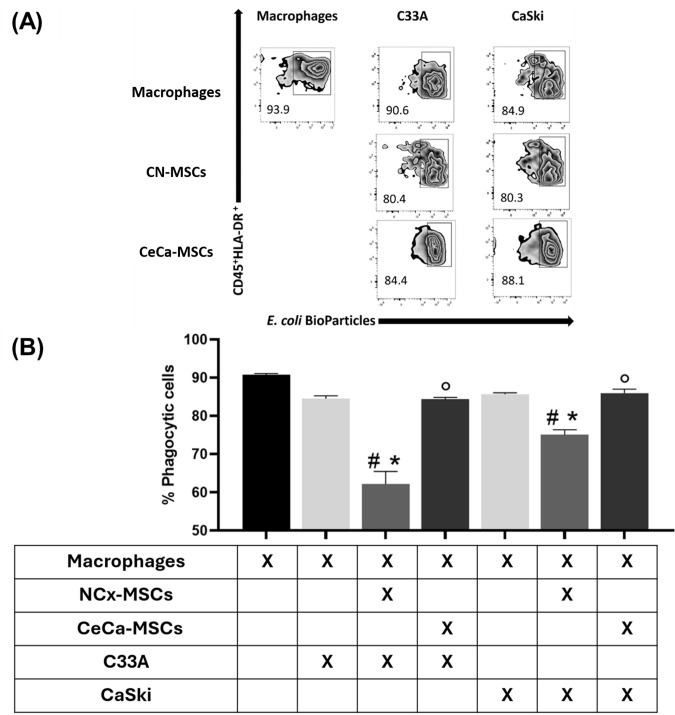
Macrophages from cocultures with TCs/NCx-MSCs decreased their phagocytic capacity. To determine the phagocytic capacity of macrophages, the macrophages obtained from cocultures of TCs/MSCs/monocytes were cultured with TCs/MSCs and labeled with pH Rodo Green E. coli BioParticles Conjugate. The percentage of phagocytic cells was determined by flow cytometry. (**A**) Zebra plots representative of the percentage of phagocytic macrophages. (**B**) Bar graphs of the percentage of macrophages with phagocytic capacity. Bar graphs represent the means with standard errors (n = 5). The Kruskal–Wallis test followed by the Mann–Whitney U post hoc test was conducted. * Significant difference with respect to the control. ^#^ Significant difference with respect to tumor line. ° Significant difference with respect to NCx-MSCs. *^, #,^ ° Significant difference, *p* < 0.05. TCs: tumor cells; C33A and CaSKi: cervical cancer cell lines; NCx-MSCs: normal cervix-derived mesenchymal stromal cells; CeCa-MSCs: cervical-cancer-derived mesenchymal stem cells.

Previous work indicates that the interaction of cells in the tumor microenvironment, such as tumor cells [10,33] and stromal cells [18], has the capacity to favor macrophage phagocytosis to eliminate apoptotic cells and promote tumor progression. In the present work, we analyzed whether TC/MSC cocultures affected the phagocytic capacity of macrophages. To determine the phagocytic capacity of macrophages, they were cocultured with TCs/MSCs and labeled with pH Rodo Green E. coli BioParticles Conjugate, which were quantified by flow cytometry. As can be seen in Figure 5, macrophages obtained from TCs/NCx-MSCs cocultures decreased the population with phagocytic capacity (C33A: 62.18% ± 3.31; CaSki: 75.12% ± 1.29) compared to those obtained from cocultures with TCs (C33A: 84.58% ± 0.67; CaSki: 85.68% ± 0.37), TCs/CeCa-MSCs (C33A: 84.36% ± 0.48; CaSki: 85.98% ± 1.03), or the control. These results show that NCx-MSCs lower the percentage of macrophages with phagocytic capacity cocultured with CaSki and C33A cell lines, which is related to the reduced ability we observed in such cocultures to polarize macrophages to an M2 phenotype.

### 3.6. NCx-MSCs Decreased Soluble IL-10 in Cocultures of Macrophages with Tumor Cells and Increased Soluble IL-6 in Those with the C33A Cell Line

**Figure 6:**
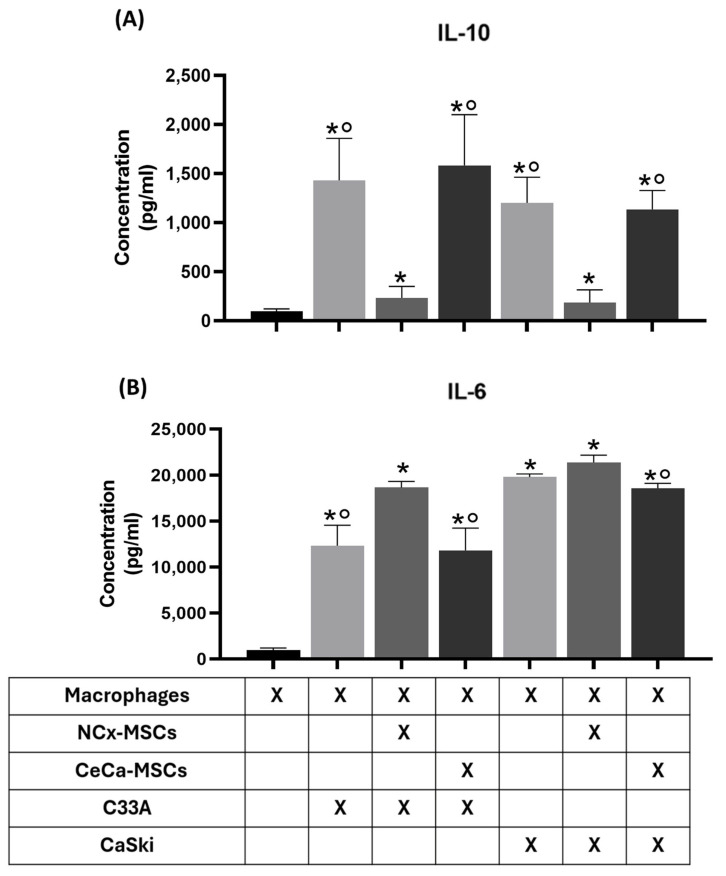
Macrophages cocultured with TCs/NCx-MSCs decreased the soluble IL-10 concentration and increased the soluble IL-6 concentration. To determine the concentration of IL-10 and IL-6 secreted into the medium, the supernatants of TC/MSC/monocyte cocultures were analyzed using a panel kit cytometry bead, and the concentration of interleukins was quantified by flow cytometry. Bar graphs of the concentration (pg/mL) of soluble IL-10 (**A**) and soluble IL-6 (**B**) in the supernatant of the indicated cultures. Bar graphs represent the means with standard errors (n = 5). The Kruskal–Wallis test followed by the Mann–Whitney U post hoc test was conducted. * Significant difference with respect to the control. ° Significant difference with respect to NCx-MSCs. *, ° Significant difference, *p* < 0.05. TCs: tumor cells; C33A and CaSKi: cervical cancer cell lines; NCx-MSCs: normal cervix-derived mesenchymal stromal cells; CeCa-MSCs: cervical-cancer-derived mesenchymal stem cells.

It has been demonstrated that the extracellular secretion of IL-10 or IL-6 by CeCa-TCs [14] or IL-10 [21] by CeCa-MSCs promotes tumor development. In our results, we observed that the coculture of macrophages with TCs/CeCa-MSCs favored the M2 phenotype, which can release anti-inflammatory cytokines with pro-tumorigenic activity. We decided to analyze the presence of soluble inflammatory cytokines in the supernatants from cocultures of macrophages with TCs/MSCs. To determine the concentration of IL-10 and IL-6 secreted into the medium, the supernatants of the cocultures were analyzed using panel kit cytometry beads, and the concentration of interleukins was quantified by flow cytometry. Our results show that macrophages cocultured with TCs/NCx-MSCs (C33A: 234.49 ± 17.21 pg/mL; CaSki: 187.36 ± 27.24 pg/mL) significantly (*p* < 0.05) decreased the concentration of soluble IL-10 when compared to the concentration observed in TCs (C33A: 1432.89 ± 426.19 pg/mL; CaSki: 1202.40 ± 261.52 pg/mL) or TCs/CeCa-MSCs (C33A: 1584.98 ± 516.86 pg/mL; CaSki: 1136.55 ± 192.52 pg/mL) (Figure 6A). Interestingly, macrophages in coculture with TCs/NCx-MSCs (C33A: 18,678.60 ± 656.16 pg/mL; CaSki: 21,400.72 ± 778.55 pg/mL) significantly (*p* < 0.05) increased the concentration of soluble IL-6 compared to those with TCs (C33A: 12,353.78 ± 2216.91 pg/mL) or TCs/CeCa-MSCs (C33A: 11,848 ± 656.16 pg/mL; CaSki: 18,587 ± 546.79 pg/mL) (Figure 6B). Our results indicate that the concentration of IL-10 decreased in cocultures of macrophages with NCx-MSCs compared to those with both cell lines and TCs/CeCa-MSCs, while the concentration of IL-6 increased in cocultures with NCx-MSCs/C33A compared to those in the presence of C33A alone and C33A/CeCa-MSCs, which suggests a decreased macrophage polarization toward an M2 phenotype in the presence of NCx-MSCs, which is consistent with our previous results obtained in such cocultures.

## 4. Discussion

Macrophages are part of the innate immune system, and their biological functions are associated with phagocytic capacity for pathogen elimination, wound regeneration, and angiogenesis [34,35]. It has been described that, in the tumor microenvironment, macrophages can be up to 50% of the infiltrating cells and are characterized by their polarization to an M2 phenotype in response to cells from the generated microenvironment, such as tumor cells and tumor-derived MSCs present [7,8,18,24,34]. To date, there is no study that has evaluated whether the interaction of tumor cells (TCs) with MSCs in the absence of external molecules influences macrophage polarization. The present work provides evidence that TC–MSC interaction has a different effect on macrophage polarization depending on whether the MSCs come from normal cervical tissue or tumor tissue.

In our study, we did not find that TC–MSC interaction decreased the expression of M1 markers; it has been described that HPV+ tumor cells decrease macrophage polarization only in the presence of LPS, a molecule that promotes M1 polarization [36]. On the other hand, our results show that TC/CeCa-MSC cocultures did not increase CD163 expression in macrophages compared to TC. Unlike other authors, in the present work, we did not use previously differentiated and/or polarized macrophages; similarly, it has been described that the effect is observed after 4 days of coculture, and Sanchez-Reyes et al. observed that the increase in CD163 expression in differentiated macrophages induced by C33A was detectable after 6 days [6,23,24]. Interestingly, we report that the coculture of TCs/NCx-MSCs decreased CD163 expression in macrophages. Several studies have reported that MSCs can have a proinflammatory (1) or anti-inflammatory (2) phenotype depending on their origin as well as the activation of TLRs by PAMPs and DAMPs; therefore, NCx-MSCs could have a predisposition to phenotype 1 derived from the characteristics of the source of procurement [37,38].

The secretion of IDO, IL-4, and IL-10 by macrophages has been described as a characteristic of the M2 phenotype [39,40,41], so to assess this, we analyzed the intracellular expression of these molecules. The results we obtained show that IDO expression in macrophages was favored only in CaSki/MSCs cocultures. The relationship of IDO activation to poor clinicopathological parameters and to worse survival in CeCa patients has been suggested [42]. On the other hand, macrophages increased intracellular IL-4 and IL-10 expression when in the presence of CaSki/CeCa-MSCs, similar to what was observed with TCs [17,27] or with MSCs [43] in separate tests. Furthermore, IL-4 expression by macrophages could amplify their functional capacities according to the M2 phenotype. It is worth noting that macrophages obtained from TC/NCx-MSC coculture decreased their IL-4 and IL-10 expression, which could suggest the possibility that these MSCs polarize macrophages towards a proinflammatory phenotype typical of MSC1 phenotype [44].

Several studies have indicated that IL-4 and IL-10 generate anergy and suppression of CD3 T lymphocyte proliferation [45,46]. In our results, we found that TCs/CeCa-MSCs increased these intracellular molecules in macrophages, and these decreased the proliferation of CD3+, CD4+, and CD8+ T lymphocytes, in contrast to macrophages generated by the interaction of TCs/NCx-MSCs that express less IL-4 and IL-10, which are related to decreasing CD28 expression, as well as limiting the secretion of cytokines such as IL-2, IFN-γ, IL-5, and TNF-α by T lymphocytes, thus suppressing their proliferation and effector action [45,46], suggesting that their activity is possibly proinflammatory.

It has been reported that M2 macrophages expressing IL-10 have the capacity to induce the generation of T lymphocytes with the CD3+CD4+C25+FOXP3+ phenotype [47]. Like our results, the presence of CD163+ macrophages has been related to a higher infiltration of FOXP3+ T lymphocytes and a worse prognosis for patients with CeCa [48]. In our work, we found that TCs/NCx-MSCs decreased the percentage of generated regulatory T lymphocytes compared to TCs or TCs/CeCa-MSCs. Previous reports indicate that NCx-MSCs do not have an immunosuppressive effect when stimulating TCs on T lymphocytes [21]. It has also been reported that TCs or MSCs favor the generation of the regulatory T phenotype in lymphocytes in in vitro systems and in murine models [49,50].

Another feature of macrophages with an M2 phenotype is their phagocytic capacity [51]. Here, we report that macrophages obtained from the TC/NCx-MSC cocultures decreased their population with phagocytic capacity. This is similar to previous results obtained by our working group, where NCx-MSCs decreased the population of macrophages with phagocytic capacity, with this effect being further observed in macrophages that were in an M1 polarization medium [18]. Interestingly, TC/CeCa-MSC cocultures did not increase this phagocytic capacity. TCs promote the ability to phagocytose apoptotic cells in a process called efferocytosis, which causes them to release IL-10 into the extracellular medium and suppress the adaptive inflammatory response. In turn, tumor cells express the CD47 marker, which prevents them from being recognized for phagocytosis [52,53].

Our working group has previously reported the ability of TCs from cervical cancer to express IL-10 as a form of immunoregulation [54]. In addition, MSC1 phenotypes has been reported to secrete IL-6 [55]. Our results show that macrophage cocultures with TCs/NCx-MSCs decreased IL-10 secretion while favoring IL-6 secretion. Different authors have related IL-6 expression in MSCs to its activation by TLR-4 through the recognition of PAMPs or DAMPs, with the aim of initiating an inflammatory response [38,44,55,56]. IL-6 has been reported to be related to macrophage polarization towards an M1 phenotype [26]; therefore, this could be why the activity of macrophages cocultured with TCs/NCx-MSCs previously reported presents a decrease in M2 polarization.

## 5. Conclusions

The present study is the first to show the effect of the interaction between TCs/CeCa-MSCs and TCs/NCx-MSCs on macrophage polarization, demonstrating that NCx-MSCs, in contrast to CeCa-MSCs, decrease CD163 expression in macrophages cocultured with CaSki cell line and that such macrophages generated from TCs/NCx-MSCs have a lower expression of IL-4 and IL-10, which results in a lower capacity to decrease T cell proliferation, as well as a lower capacity to generate CD4+C25+FOXP3+ regulatory T cell populations. In addition, we demonstrated that these macrophages, as opposed to those from cultures in the presence of CeCa-MSCs, present a lower population with phagocytic capacity, and we found that there is a lower concentration of IL-10 and a higher concentration of soluble IL-6, mainly in the presence of the C33A cell line, in the medium of such cocultures. Based on our results in an in vitro system, we suggest that CeCa-MSCs in communication with tumor cells induce M2 macrophage polarization, which favors pro-tumor activity, while NCx-MSCs in the presence of tumor cells present a proinflammatory phenotype, which could be associated with MSC1 phenotype, thus favoring the M1 polarization of macrophages, suggesting anti-tumoral activity. It is necessary to know which molecules are involved in the anti-inflammatory profile of CeCa-MSCs and in the proinflammatory profile of NCx-MSCs and how such molecules affect the behavior of tumor cells; related experiments are being planned for future analysis. Furthermore, due to the complexity of the tumor microenvironment, the analysis of other immune populations involved in the development of tumor cells, such as B cells, NK cells, and dendritic cells, among others, must be addressed in experimental studies to complement the complex network of cells, cytokines, and extracellular matrix proteins that promote the progression of this type of cancer. Our research group is considering experimental studies related to this in the future.

**Figure 7:**
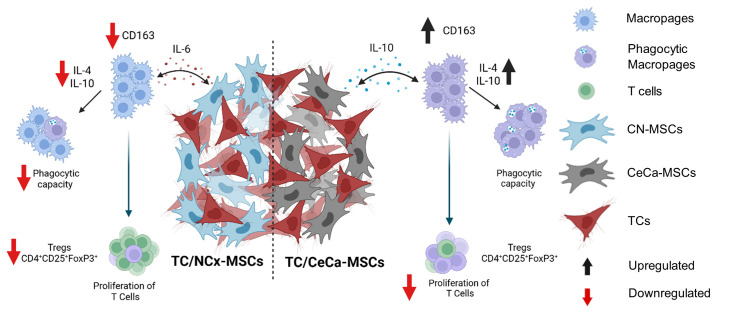
Coculture of TCs/CeCa-MSCs promote higher in vitro M2 polarization of macrophages than coculture of TCs/NCx-MSCs. Schematic diagram of the in vitro mechanism of macrophage polarization towards the M2 phenotype mediated by the cocultures of TCs/NCx-CeCa and TCs/CeCa-MSCs, in terms of the modification of surface markers, intracellular molecules, phagocytosis capacities, and generation of Tregs lymphocytes, as well as the decrease in the proliferation of T lymphocytes by macrophages. The diagram shows the presence of cytokines in the supernatant that could play a role in macrophage polarization. Created in BioRender. Bautista, E. (2025). https://BioRender.com/3r6wzlj (accessed on 20 June 2024).

In summary, our findings show that the interaction between TCs and CeCa-MSCs favors the M2 polarization of macrophages that show a high immunosuppressive capacity. This study suggests that cell communication between TCs and MSCs present in cervical tumors could favor tumor growth through the polarization of macrophages with high immunosuppressive potential (Figure 7).

## Data Availability

The original contributions presented in this study are included in the article; further inquiries can be directed to the corresponding author.
